# Supplementary figures and images for: Similar functional composition of fish assemblages despite contrasting levels of habitat degradation on shallow Caribbean coral reefs
Source: PLoS One. 2023 Dec 27;18(12):e0295238. doi: 10.1371/journal.pone.0295238 (PMC10752542; doi:10.1371/journal.pone.0295238)

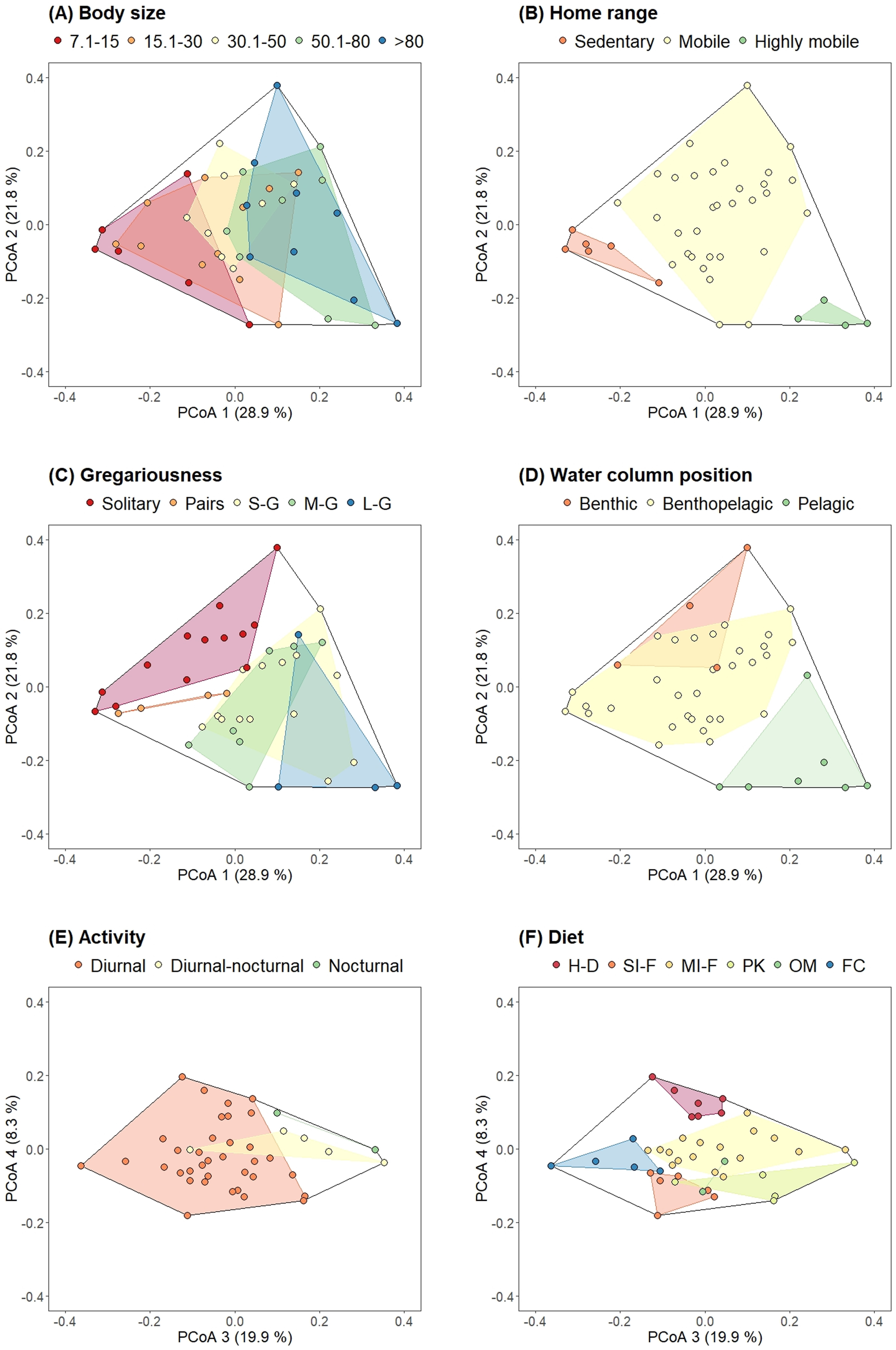

Supplement: S1 Fig — Figures A-D display the convex hulls for body size, home range, gregariousness, and water column position traits in PCoA 1 and PCoA 2, while figures e-f display the convex hulls for activity and diet along PCoA 3 and PCoA 4. In (C) gregariousness: S-G (small groups), M-G (medium groups), L-G (large groups); (F) Diet: H-D (herbivores-detritivores), SI-F (sessile invertebrate feeders), MI-F (mobile benthic invertebrate feeders), PK (planktivores), OM (omnivores), and FC (piscivores). (TIF) [file pone.0295238.s001.tif]

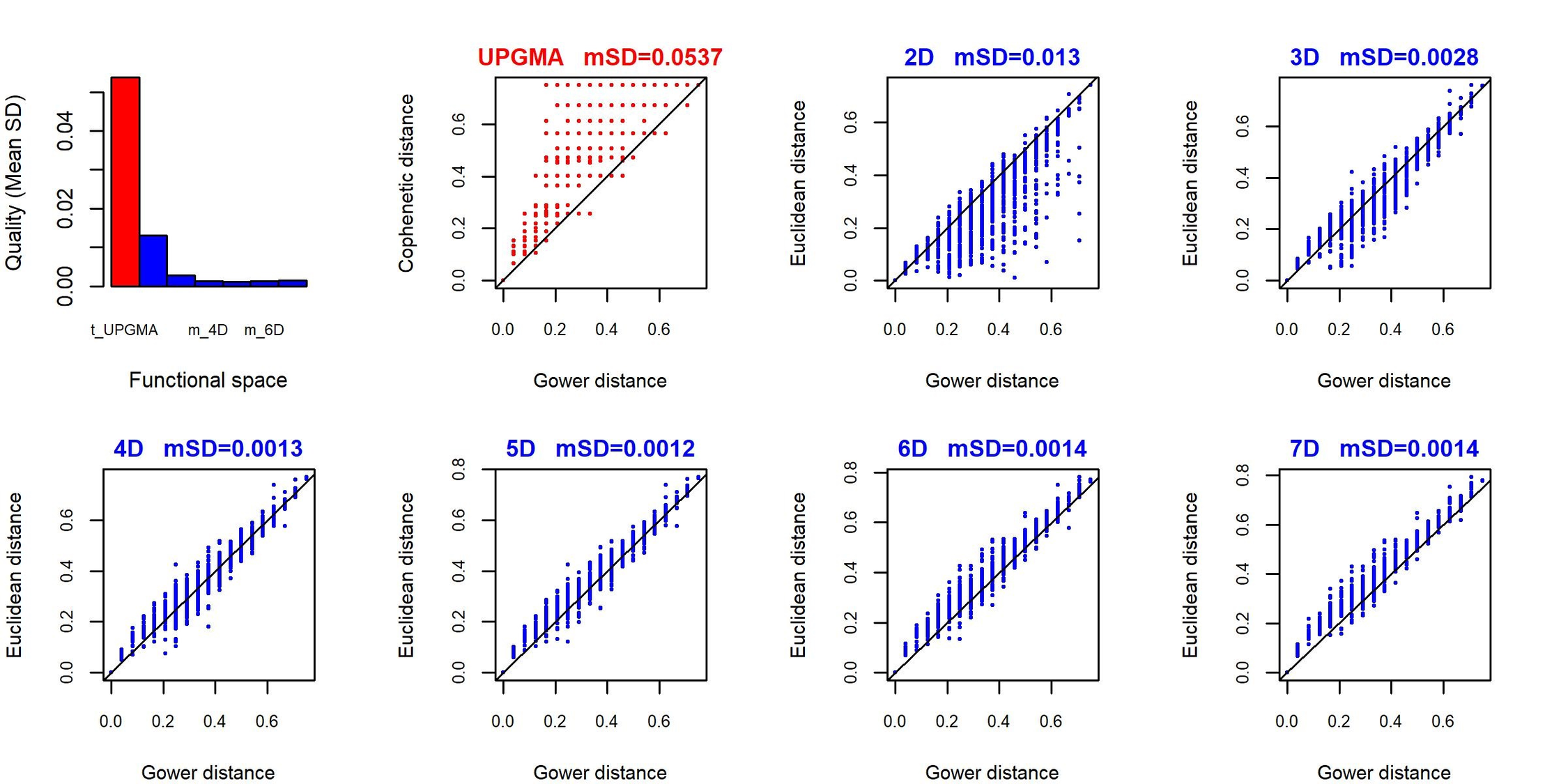

Supplement: S2 Fig — The comparison with an UPGMA dendrogram is also shown. The mean squared deviation (mSD) is used to assess the quality of the functional space. (TIF) [file pone.0295238.s002.tif]

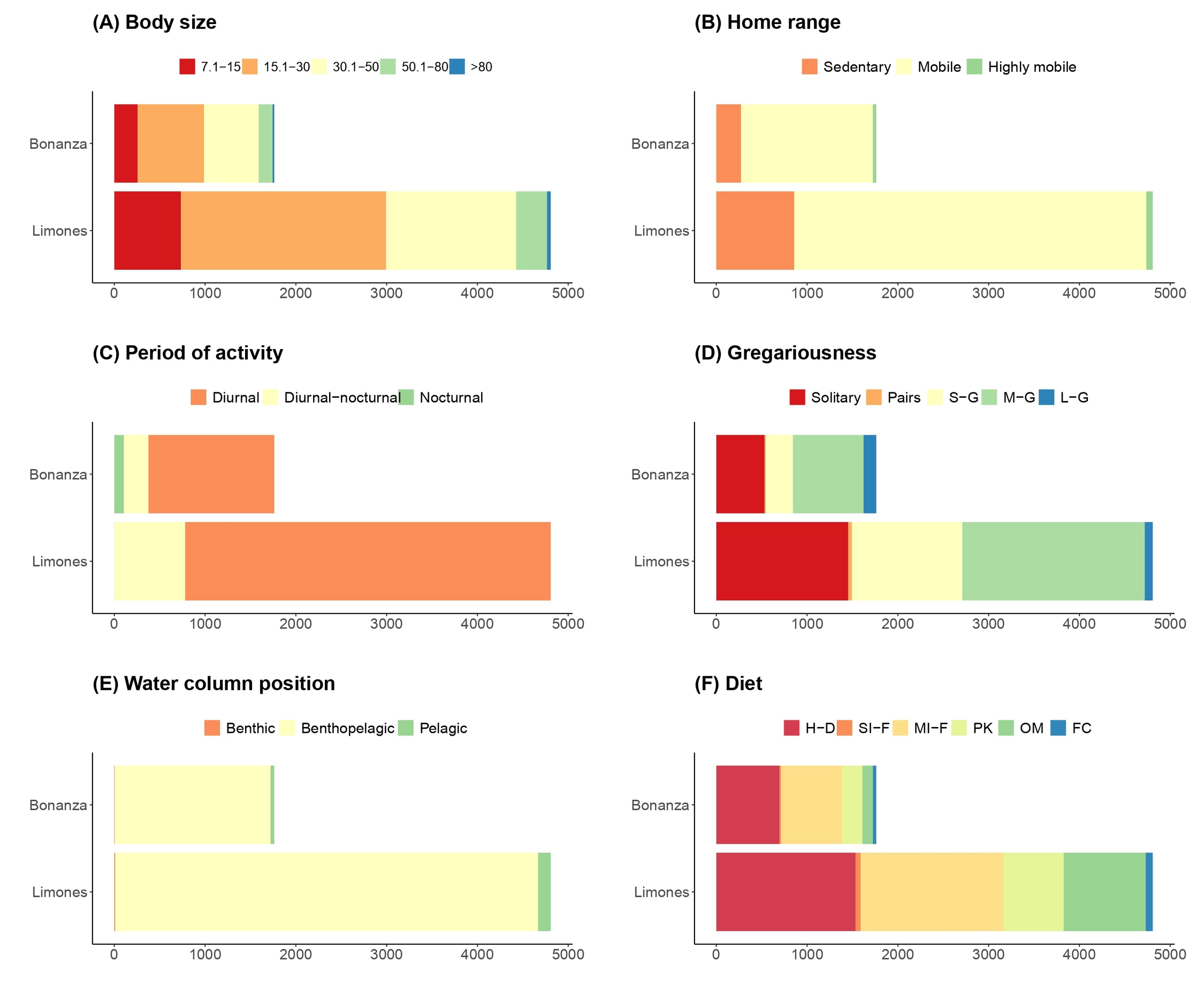

Supplement: S3 Fig — Bar charts show the fish absolute abundance of each trait category calculated from the community-level weighted means of trait values (CWM) for a set of 68 fish species. In (d) gregariousness: S-G (small groups), M-G (medium groups), L-G (large groups); (f) Diet: H-D (herbivores-detritivores), SI-F (sessile invertebrate feeders), MI-F (mobile benthic invertebrate feeders), PK (planktivores), OM (omnivores), and FC (piscivores). (TIF) [file pone.0295238.s003.tif]

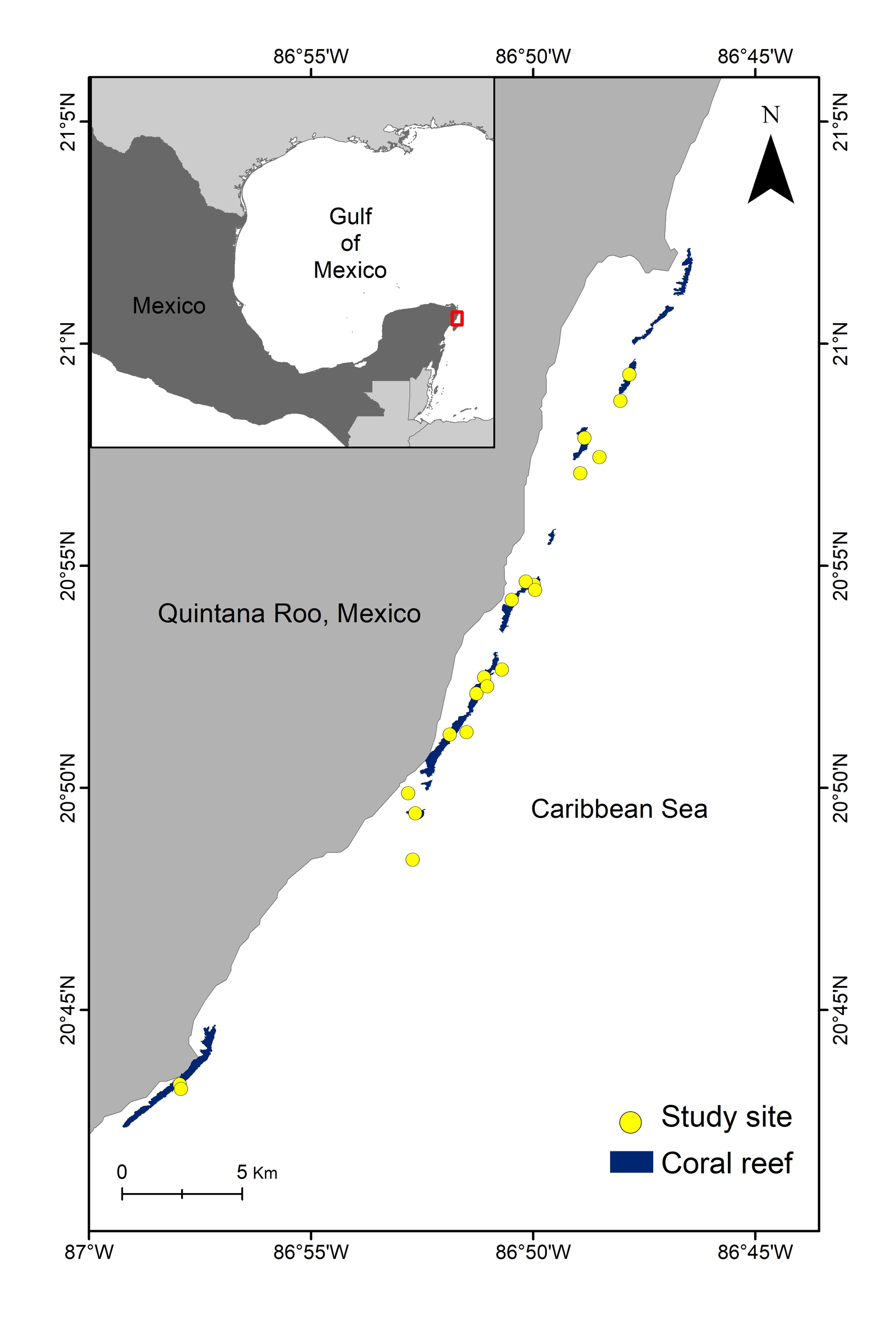

Supplement: S4 Fig — The study sites are in the northern portion of the Mexican Caribbean (including the Puerto Morelos reef system). (TIF) [file pone.0295238.s004.tif]

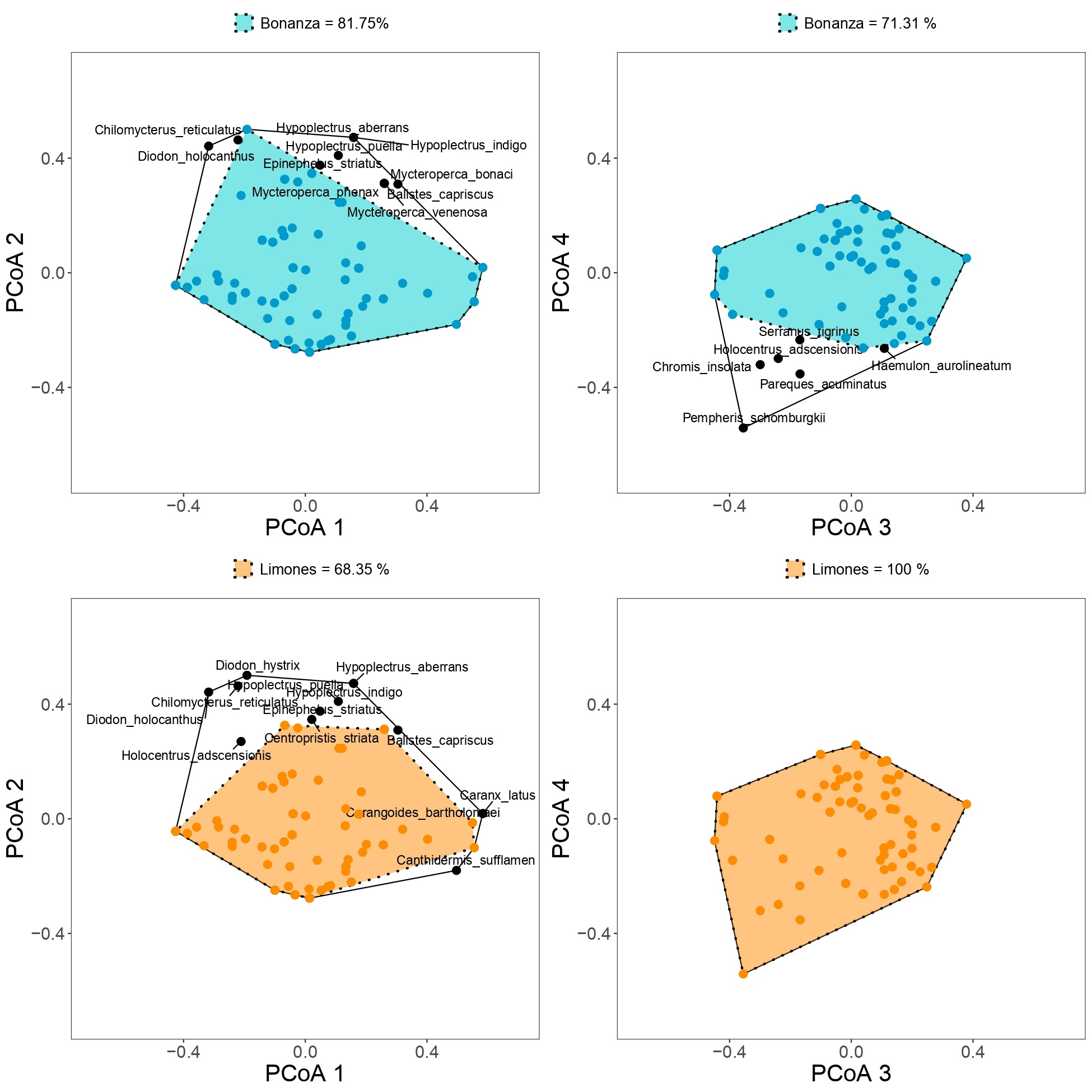

Supplement: S5 Fig — Multidimensional functional space filled by fish assemblages in Bonanza (upper panel) and Limones (bottom panel). The black polygons represent the functional space occupied by all fish species present on 20 reef sites of the Puerto Morelos reef system. The turquoise-filled polygon represents the functional space occupied by fish trait combinations in Bonanza while the orange-filled polygon represents the functional space occupied by the fish trait combinations in Limones. Black points depict the reef fish species outside of the functional space covered by the polygons of Bonanza and Limones. (TIF) [file pone.0295238.s005.tif]
