# Supplementary material for: Similar functional composition of fish assemblages despite contrasting levels of habitat degradation on shallow Caribbean coral reefs
Source: PLoS One. 2023 Dec 27;18(12):e0295238. doi: 10.1371/journal.pone.0295238 (PMC10752542; doi:10.1371/journal.pone.0295238)
Supplement: S1 Table — We selected six traits that describe several facets of fish ecology and are available for many species of tropical regions. These traits were employed to perform the functional diversity indices and they have been shown to have ecological implications for ecosystem functioning and a range of responses and effects by the coral reef fishes against habitat changes. (PDF) [file pone.0295238.s006.pdf]

Supporting information

S1 Table. Functional traits selected for this study to explore changes in functional structure of Caribbean reef fishes. We selected six traits that describe several facets of fish ecology and are available for many species of tropical regions. These traits were employed to perform the functional diversity indices and they have been shown to have ecological implications for ecosystem functioning and a range of responses and effects by the coral reef fishes against habitat changes.

Trait	Ecological implications	Response/effect on habitat*
**Body size**	Body size acts as a primary driver of ecological processes that directly impact almost all basic anatomical, physiological, and behavioral parameters [1]. Body size also captures the variation of functions relating to feeding, movement, home range and energetic requirements [2].<br>Body size has been closely tied to ecosystem functions such as nutrient cycling [3], bioerosion [4] or growth [5]. Body size determines energy needs through the amount of energy required per unit of body mass [6] and small fish species are a major contributor to reef energy turnover [7].	The consequences of coral habitat degradation are particularly severe on small body size individuals [9]. Many fishes that feed or shelter among live coral colonies are small bodied, and disturbances that reduce coral cover and their rugosity have detrimental effect on their abundance [10]. In addition, habitat loss also results in fewer small-bodied juveniles and prey that replenish stocks and provide dietary resources for predatory target species [11]. In some cases, the decline of larger bodied fish relates partially to a higher dependence on live coral and associated structure

	Body size also affects the fish ability to swim. For example, larger fishes are expected to be faster and have greater endurance than small ones, but small fishes have better maneuverability and can thus move in topographically complex environments such as coral reefs [8].	during the early life history, when fish are smaller [12].<br>Thus, reef habitat availability of shelter of different sizes has the potential to control the abundance of fish body sizes that can use available holes, suggesting that the absolute availability of holes for refuge-dependent, small, and mid-sized fish declines as the reef degrades, and that more uniform fish size distributions are associated with homogenous versus heterogeneous and complex environments [13,14].
**Home range**	Home range is influenced by fish mobility and determines energy needs, with mobile species requiring a lot of energy by mass unit compared with sedentary species [6].<br>Home range also affects the spatial extent at which fishes control their resources and transfer nutrients, especially between habitats around reefs [6].	Traditionally, the study of species home range has been used to identify key components of habitat such as important foraging and shelter areas as well as migration paths [16]. Thus, declines in the abundance of fishes following habitat degradation may reflect the large-scale migration of fishes to nearby and relatively unaffected habitats but also can limit the ability of certain species to find

	Some coral reef fishes move daily between sites used for feeding and those used for reproduction or for resting. Often, these activities occur in different habitat types and species with mobility divided between two habitat types tend to have larger home ranges than species with only a single habitat type [15].<br>Home range scales positively with body size, and the degree of exclusivity habitat use declines with increasing body size due to the metabolic scaling of energy acquisition and as larger fishes tend to also increase in mobility [13].	alternative habitats depending on their home range [9]. In addition, some reef fish exhibit ontogenetic shifts in habitat use and may relocate their home ranges in response to habitat changes [15].
**Period of activity**	Period of activity or diel activity has implications on the trophic role a species plays in the food web through both bottom-up controls (i.e., the set of resources it can target) and top-down controls (i.e., the susceptibility it has for being preyed upon) [6].<br>Diurnal fish are active in the day, retreating to the reef matrix at night,	Diel period variation in habitat usage by fish remains understudied. However, some studies have found that habitat associations displayed by fishes during the day are not always maintained at night [18]. As dusk approaches, some species migrate out onto areas such as deep reefs, rhodolith, sand, and seagrass habitats

	when the nocturnal fish emerge [17]. These diurnal and nocturnal migrations may be driven by feeding or reproductive behavior and further influenced by the presence of predators [18].<br>Period of activity also describes temporal fish species turnover within the reef fish assemblages [19]. For example, many grunts (Haemulidae) rest during the day on coral reefs but feed at night over soft substrates [15].	to feed at night. For example, nocturnally active Haemulidae and Lutjanidae migrate from sheltered sites during the day to seagrass beds at night [20,21]. Therefore, day and night activity patterns provide an ecologically meaningful unit for scaling the environment in habitat-use studies and are important in defining suitable seascapes for some fish species that move across spatially heterogenous patches.
**Gregariousness**	Gregariousness or schooling influences predation vulnerability, nutrient cycling, and resource depletion [6]. Schooling behavior describes species social strategies that minimize predation and energetic costs while feeding [19]. Particularly, many fishes exhibit a schooling behavior when young, whereas older individuals are solitary [22]. This indicates the presence of a range of schooling types, depending on age, physiological state, and factors such as habitat type,	The appearance / absence of schooling behavior in fishes of the same species may be caused by changes in the ecological conditions. There is a clear tendency to schooling in homogeneous habitats but an individual mode of life in more complex heterogeneous habitats [22]. Particularly, non-aggregators (e.g., non-social, and often territorial species) may occur at high densities where resources and shelter are abundant, but do not shoal [23]. In

	activity, environmental conditions, etc. Most nocturnal species get away predation from active predators during the day (e.g., finding shelter in preserved habitat).	contrast, refuge-based aggregators (e.g., damselfishes) form shoals because of the spatial patchiness of refuges such as branching corals, while social aggregators (e.g., many wrasses) form high-density aggregations independent of refuge availability, apparently yielding antipredator benefits [24].
**Position in water column**	Position in the water column is critical to determining fish ecological niche and influences species distributions, mobility, nutrient transfer between vertical strata, and habitat requirements [6].<br>Water column position also strongly influences vulnerability to predation [25]. For example, red snappers are known to be active, opportunistic predators consuming a variety of prey species including benthic crustaceans, reef-associated fishes, pelagic fishes, squids, and zooplankton [26].	The position in the water column in which the species usually forage is a good indicator of their swimming ability and can indicate the dependence of a species on the substrate [27]. Although water transports particles (e.g., zooplankton), maintaining water column position in high flows is energetically costly. Thus, structural complexity provides fishes with refuges against both water flow and predators [5].

**Diet**	Diet determines fish impact on ecosystem functioning through trophic interactions with other food resources, contributing to nutrient cycles [6]. Nutrient acquisition depends on diet composition, i.e., which resources are consumed, and the nutrient content of these resources [8]. The top trophic position fish on coral reefs typically have diverse fish prey drawn from all trophic levels, including the bottom of reef pyramid [28]. Likewise, numerous small-bodied, coral associated planktivorous fishes perform the transfer of pelagic nutrients to the benthic community [29].	Diet mediates habitat requirements of fishes because some resources are restricted to specific habitats [30]. For instance, some epilithic algal feeding pomacentrids also show a preference for habitats with skeletons of branching corals, whilst invertivores tend to be habitat generalists and are therefore expected to be less susceptible to habitat disturbance [10]. Reefs with greater structural complexity support longer food chains with more predator-dominated fish communities compared to flatter reefs with low coral cover [13,14]. Therefore, responses of fishes to coral loss and habitat degradation do vary with trophic roles, often showing a negative relationship, especially with coral-dependent species [12].

*The “response-and-effect framework” postulates that it is useful to determine which traits respond to environmental gradients (“response traits”) and which traits affect ecosystem processes (“effects traits”) (Hadj-Hammou et al., [31]).

References

1. Bellwood DR, Streit RP, Brandl SJ, Tebbett SB. The meaning of the term 'function' in ecology: A coral reef perspective. Funct Ecol. 2019;33: 948–961. doi:10.1111/1365-2435.13265
2. Richardson LE, Graham NAJ, Pratchett MS, Hoey AS. Structural complexity mediates functional structure of reef fish assemblages among coral habitats. Environ Biol Fishes. 2017;100: 193–207. doi:10.1007/s10641-016-0571-0
3. Allgeier JE, Layman CA, Mumby PJ, Rosemond AD. Consistent nutrient storage and supply mediated by diverse fish communities in coral reef ecosystems. Glob Chang Biol. 2014;20: 2459–2472. doi:10.1111/gcb.12566
4. Bonaldo RM, Hoey AS, Bellwood DR. The Ecosystem Roles of Parrotfishes on Tropical Reefs. Oceanogr Mar Biol. 2014; 81–132. doi:10.1201/b17143-3
5. Morais RA, Bellwood DR. Global drivers of reef fish growth. Fish Fish. 2018;19: 874–889. doi:10.1111/faf.12297
6. Mouillot D, Villéger S, Parravicini V, Kulbicki M, Arias-González JE, Bender M, et al. Functional over-redundancy and high functional vulnerability in global fish faunas on tropical reefs. Proc Natl Acad Sci U S A. 2014;111: 13757–13762. doi:10.1073/pnas.1317625111
7. Brandl SJ, Rasher DB, Côté IM, Casey JM, Darling ES, Lefcheck JS, et al. Coral reef ecosystem functioning: eight core processes and the role of biodiversity. Front Ecol Environ. 2019;17: 445–454. doi:10.1002/fee.2088
8. Villéger S, Brosse S, Mouchet M, Mouillot D, Vanni MJ. Functional ecology of fish: current approaches and future challenges. Aquat Sci. 2017;79: 783–801. doi:10.1007/s00027-017-0546-z
9. Wilson SK, Graham NAJ, Pratchett MS, Jones GP, Polunin NVC. Multiple disturbances and the global degradation of coral reefs: Are reef fishes at risk or resilient? Glob Chang Biol.

2006;12: 2220–2234. doi:10.1111/j.1365-2486.2006.01252.x

10. Wilson SK, Fisher R, Pratchett MS, Graham NAJ, Dulvy NK, Turner RA, et al. Exploitation and habitat degradation as agents of change within coral reef fish communities. Glob Chang Biol. 2008;14: 2796–2809. doi:10.1111/j.1365-2486.2008.01696.x

11. Wilson SK, Fisher R, Pratchett MS, Graham NAJ, Dulvy NK, Cakacaka A, et al. Habitat degradation and fishing effects on the size structure of coral reef fish communities Published by : Wiley on behalf of the Ecological Society of America Stable URL : http://www.jstor.org/stable/27797819 REFERENCES Linked references are available o. Ecol Appl. 2010;20: 442–451.

12. Pratchett MS, Hoey AS, Wilson SK. Reef degradation and the loss of critical ecosystem goods and services provided by coral reef fishes. Curr Opin Environ Sustain. 2014;7: 37–43. doi:10.1016/j.cosust.2013.11.022

13. Alvarez-Filip L, Gill JA, Dulvy NK. Complex reef architecture supports more small-bodied fishes and longer food chains on Caribbean reefs. Ecosphere. 2011;2: art118. doi:10.1890/es11-00185.1

14. Nash KL, Graham NAJ, Wilson SK, Bellwood DR. Cross-scale Habitat Structure Drives Fish Body Size Distributions on Coral Reefs. Ecosystems. 2013;16: 478–490. doi:10.1007/s10021-012-9625-0

15. Kramer DL, Chapman MR. Implications of fish home range size and relocation for marine reserve function. Environmental Biology of Fishes. 1999. pp. 65–79. doi:10.1023/a:1007481206399

16. Nash KL, Welsh JQ, Graham NAJ, Bellwood DR. Home-range allometry in coral reef fishes: comparison to other vertebrates, methodological issues and management implications. Oecologia. 2015;177: 73–83. doi:10.1007/s00442-014-3152-y

17. MacNeil MA, Connolly SR. 12 Multi-scale patterns and processes in reef fish abundance. Ecol fishes coral reefs. 2015; 116.

18. Harvey ES, Butler JJ, McLean DL, Shand J. Contrasting habitat use of diurnal and nocturnal fish assemblages in temperate Western Australia. J Exp Mar Bio Ecol. 2012;426–427: 78–86. doi:10.1016/j.jembe.2012.05.019

19. Rincón-Díaz MP, Pittman SJ, Arismendi I, Heppell SS. Functional diversity metrics detect spatio-temporal changes in the fish communities of a Caribbean marine protected area. Ecosphere. 2018;9. doi:10.1002/ecs2.2433

20. Hammerschlag N, Serafy JE. Nocturnal fish utilization of a subtropical mangrove-seagrass ecotone. Mar Ecol. 2010;31: 364–374. doi:10.1111/j.1439-0485.2009.00337.x

21. Hitt S, Pittman SJ, Nemeth RS. Diel movements of fishes linked to benthic seascape structure in a caribbean coral reef ecosystem. Mar Ecol Prog Ser. 2011;427: 275–291. doi:10.3354/meps09093

22. Pavlov DS, Kasumyan AO. Patterns and mechanisms of schooling behaviour in fish: A review. J Ichthyol. 2000;40: 163–231.

23. White JW, Samhouri JF, Stier AC, Wormald CL, Hamilton SL, Sandin SA. Synthesizing mechanisms of density dependence in reef fishes: behavior, habitat configuration, and observational scale. Ecology. 2010;91: 1949–1961.

24. White JW, Warner RR. Behavioral and energetic costs of group membership in a coral reef fish. Oecologia. 2007;154: 423–433. doi:10.1007/s00442-007-0838-4

25. Green SJ, Côté IM. Trait-based diet selection: Prey behaviour and morphology predict vulnerability to predation in reef fish communities. J Anim Ecol. 2014;83: 1451–1460. doi:10.1111/1365-2656.12250

26. Williams-Grove LJ, Szedlmayer ST. Depth preferences and three-dimensional movements of red snapper, Lutjanus campechanus, on an artificial reef in the northern Gulf of Mexico. Fish Res. 2017;190: 61–70.

27. Luiz OJ, Allen AP, Robertson DR, Floeter SR, Madin JS. Seafarers or castaways: Ecological traits associated with rafting dispersal in tropical reef fishes. J Biogeogr. 2015;42: 2323–

2333. doi:10.1111/jbi.12574

28. Graham NAJ, McClanahan TR, MacNeil MA, Wilson SK, Cinner JE, Huchery C, et al. Human Disruption of Coral Reef Trophic Structure. Curr Biol. 2017;27: 231–236. doi:10.1016/j.cub.2016.10.062

29. Brandl SJ, Emslie MJ, Ceccarelli DM. Habitat degradation increases functional originality in highly diverse coral reef fish assemblages. Ecosphere. 2016;7. doi:10.1002/ecs2.1557

30. Brandl SJ, Bellwood DR. Morphology, sociality, and ecology: Can morphology predict pairing behavior in coral reef fishes? Coral Reefs. 2013;32: 835–846. doi:10.1007/s00338-013-1042-0

31. Hadj-Hammou J, Mouillot D, Graham NAJ. Response and Effect Traits of Coral Reef Fish. Front Mar Sci. 2021;8: 1–14. doi:10.3389/fmars.2021.640619
