# Supplementary material for: Similar functional composition of fish assemblages despite contrasting levels of habitat degradation on shallow Caribbean coral reefs
Source: PLoS One. 2023 Dec 27;18(12):e0295238. doi: 10.1371/journal.pone.0295238 (PMC10752542; doi:10.1371/journal.pone.0295238)
Supplement: S2 Table — Species traits were compiled by Quimbayo et al. (2021) and were coded into several categories according to Mouillot et al. (2014). Body size: 0–7 cm (s1), 7.1–15 cm (s2), 15.1–30 cm (s3), 30.1–50 cm (s4), 50.1–80 cm (s5), and >80 cm (s6). Home range: sedentary-territorial species (sed), mobile species (mob), and very mobile species (vmob). Period of activity: diurnal species (day), diurnal-nocturnal species (both), and nocturnal species (night). Gregariousness: solitary (sol), pairs (pair), small groups (smallg), medium groups (medg), and large groups (largeg). Position in water column: benthic (bottom), benthopelagic (low), pelagic (high). Diet: herbivore-detritivore (hd), macroalgal feeder (hm), sessile invertebrates (is), mobile benthic invertebrates (im), planktonic (pk), omnivore (om), and piscivore (fc). (PDF) [file pone.0295238.s007.pdf]

Supporting information

S2 Table. List of 68 species and 21 families of reef fishes recorded in Bonanza and Limones and their functional traits values. Species traits were compiled by Quimbayo et al. (2021) and were coded into several categories according to Mouillot et al. (2014). Body size: 0-7 cm (s1), 7.1-15 cm (s2), 15.1-30 cm (s3), 30.1-50 cm (s4), 50.1-80 cm (s5), and >80 cm (s6). Home range: sedentary-territorial species (sed), mobile species (mob), and very mobile species (vmob). Period of activity: diurnal species (day), diurnal-nocturnal species (both), and nocturnal species (night). Gregariousness: solitary (sol), pairs (pair), small groups (smallg), medium groups (medg), and large groups (largeg). Position in water column: benthic (bottom), benthopelagic (low), pelagic (high). Diet: herbivore-detritivore (hd), macroalgal feeder (hm), sessile invertebrates (is), mobile benthic invertebrates (im), planktonic (pk), omnivore (om), and piscivore (fc).

**Family**	**Species**	**Body size**	**Home range**	**Period of activity**	**Gregariousness**	**Water column position**	**Diet**
**Acanthuridae**	*Acanthurus tractus*	s4	mob	day	medg	low	hd
	Acanthurus chirurgus	s4	mob	day	medg	low	hd
	Acanthurus coeruleus	s4	mob	day	medg	low	hd
**Aulostomidae**	*Aulostomus maculatus*	s6	mob	day	smallg	low	fc
**Balistidae**	*Balistes vetula*	s5	mob	day	sol	low	im
	Canthidermis sufflamen	s5	vmob	day	smallg	high	pk
**Carangidae**	*Caranx latus*	s6	vmob	day	largeg	high	fc
	Caranx ruber	s5	vmob	day	largeg	high	fc
**Chaetodontidae**	*Chaetodon capistratus*	s2	sed	day	pair	low	is
	Chaetodon ocellatus	s3	sed	day	pair	low	is
	Chaetodon striatus	s3	sed	day	pair	low	is
**Diodontidae**	*Diodon hystrix*	s6	mob	both	sol	bottom	im

**Gerreidae**	*Eucinostomus gula*	s3	mob	night	largeg	low	im
**Haemulidae**	*Anisotremus surinamensis*	s5	mob	day	smallg	low	im
	Anisotremus virginicus	s4	mob	day	smallg	low	im
	Haemulon carbonarium	s4	mob	both	medg	low	im
	Haemulon chrysargyreum	s3	mob	both	medg	low	im
	Haemulon flavolineatum	s3	mob	both	medg	low	im
	Haemulon plumierii	s5	mob	both	medg	low	im
	Haemulon sciurus	s4	mob	both	medg	low	im
	Haemulon striatum	s3	mob	both	medg	low	im
	Haemulon vittatum	s3	mob	day	largeg	high	pk
**Labridae**	*Bodianus rufus*	s4	mob	day	sol	low	im
	Halichoeres bivittatus	s4	mob	day	sol	low	im
	Halichoeres garnoti	s3	mob	day	sol	low	im
	Halichoeres maculipinna	s3	mob	day	sol	low	im
	Halichoeres poeyi	s3	mob	day	sol	low	im
	Halichoeres radiatus	s5	mob	day	sol	low	im
	Lachnolaimus maximus	s6	mob	day	sol	low	im
	Thalassoma bifasciatum	s3	mob	day	medg	low	pk
**Lutjanidae**	*Lutjanus analis*	s6	mob	day	smallg	low	im
	Lutjanus apodus	s5	mob	day	smallg	low	im
	Lutjanus griseus	s6	mob	day	smallg	low	im
	Lutjanus mahogoni	s4	mob	day	smallg	low	im
	Ocyurus chrysurus	s6	mob	day	smallg	high	im
**Monacanthidae**	*Cantherhines pullus*	s3	mob	day	sol	bottom	is
**Mullidae**	*Mulloidichthys martinicus*	s4	mob	both	medg	low	im
	Pseudupeneus maculatus	s3	mob	day	smallg	low	im
**Ostraciidae**	*Lactophrys bicaudalis*	s4	mob	day	sol	low	im
	Lactophrys triqueter	s4	mob	day	sol	low	im
**Pempheridae**	*Pempheris schomburgkii*	s2	sed	both	medg	low	pk
**Pomacanthidae**	*Holacanthus ciliaris*	s4	mob	day	sol	low	is
	Holacanthus tricolor	s4	mob	day	sol	low	is
	Pomacanthus arcuatus	s5	mob	day	pair	low	is
	Pomacanthus paru	s4	mob	day	pair	low	is
**Pomacentridae**	*Abudefduf saxatilis*	s3	mob	day	smallg	low	om

	Chromis cyanea	s2	mob	day	medg	high	pk
	Chromis multilineata	s3	mob	day	largeg	high	pk
	Microspathodon chrysurus	s3	sed	day	sol	low	hd
	Stegastes adustus	s2	sed	day	sol	low	hd
	Stegastes diencaeus	s2	sed	day	sol	low	hd
	Stegastes leucostictus	s2	sed	day	sol	low	hd
	Stegastes partitus	s2	sed	day	sol	low	om
	Stegastes planifrons	s2	sed	day	sol	low	hd
**Priacanthidae**	*Heteropriacanthus cruentatus*	s5	mob	night	smallg	low	im
**Scaridae**	*Sparisoma atomarium*	s3	mob	day	smallg	low	hd
	Sparisoma aurofrenatum	s3	mob	day	smallg	low	hd
	Sparisoma chrysopterum	s4	mob	day	smallg	low	hd
	Scarus iseri	s4	mob	day	smallg	low	hd
	Scarus guacamaia	s6	mob	day	smallg	low	hd
	Sparisoma rubripinne	s4	mob	day	smallg	low	hd
	Scarus vetula	s5	mob	day	smallg	low	hd
	Sparisoma viride	s5	mob	day	smallg	low	hd
**Serranidae**	*Cephalopholis cruentata*	s4	mob	day	sol	bottom	im
	Mycteroperca bonaci	s6	mob	day	sol	bottom	fc
	Mycteroperca phenax	s6	mob	day	sol	bottom	fc
**Sphyraenidae**	*Sphyraena barracuda*	s6	vmob	day	smallg	high	fc
**Tetraodontidae**	*Canthigaster rostrata*	s2	mob	day	sol	low	im

14
